# Feasibility of diffusion and probabilistic white matter analysis in patients implanted with a deep brain stimulator

**DOI:** 10.1016/j.nicl.2019.102135

**Published:** 2019-12-14

**Authors:** Jennifer Muller, Mahdi Alizadeh, Lucy Li, Sara Thalheimer, Caio Matias, Mohamed Tantawi, Jingya Miao, Mackenzie Silverman, Veronica Zhang, Grace Yun, Victor Romo, Feroze B. Mohamed, Chengyuan Wu

**Affiliations:** a Jefferson Integrated Magnetic Resonance Imaging Center, Department of Radiology, Thomas Jefferson University, Philadelphia, PA, United States; b Department of Neurosurgery, Thomas Jefferson University, Philadelphia, PA, United States; c Department of Anesthesiology, Thomas Jefferson University, Philadelphia, PA, United States

**Keywords:** Postoperative imaging, Clinical feasibility, Deep brain stimulation, Diffusion weighted imaging, Tractography, Susceptibility artifact, Parkinson's disease

## Abstract

Deep brain stimulation (DBS) for Parkinson's disease (PD) is an established advanced therapy that produces therapeutic effects through high frequency stimulation. Although this therapeutic option leads to improved clinical outcomes, the mechanisms of the underlying efficacy of this treatment are not well understood. Therefore, investigation of DBS and its postoperative effects on brain architecture is of great interest. Diffusion weighted imaging (DWI) is an advanced imaging technique, which has the ability to estimate the structure of white matter fibers; however, clinical application of DWI after DBS implantation is challenging due to the strong susceptibility artifacts caused by implanted devices. This study aims to evaluate the feasibility of generating meaningful white matter reconstructions after DBS implantation; and to subsequently quantify the degree to which these tracts are affected by post-operative device-related artifacts.

DWI was safely performed before and after implanting electrodes for DBS in 9 PD patients. Differences within each subject between pre- and post-implantation FA, MD, and RD values for 123 regions of interest (ROIs) were calculated. While differences were noted globally, they were larger in regions directly affected by the artifact. White matter tracts were generated from each ROI with probabilistic tractography, revealing significant differences in the reconstruction of several white matter structures after DBS. Tracts pertinent to PD, such as regions of the substantia nigra and nigrostriatal tracts, were largely unaffected.

The aim of this study was to demonstrate the feasibility and clinical applicability of acquiring and processing DWI post-operatively in PD patients after DBS implantation. The presence of global differences provides an impetus for acquiring DWI shortly after implantation to establish a new baseline against which longitudinal changes in brain connectivity in DBS patients can be compared. Understanding that post-operative fiber tracking in patients is feasible on a clinically-relevant scale has significant implications for increasing our current understanding of the pathophysiology of movement disorders, and may provide insights into better defining the pathophysiology and therapeutic effects of DBS.

## Highlights

• Feasibility of Diffusion and Probabilistic White Matter Analysis in Patient's Implanted with a Deep Brain Stimulator.
• Postoperative diffusion imaging analysis is feasible in patients having undergone deep brain stimulation.
• Several key diffusion metrics are able to be reconstructed postoperatively in several structures relevant to Parkinson's disease.
• Successful reconstruction of three major tracts related to Parkinson's disease progression can be achieved.
• Post-op DWI may aid in understanding the mechanisms of DBS by revealing anatomical changes that occur during treatment.
• Postoperative diffusion acquisition and analysis can be achieved using clinically feasible scanning parameters.

## Nomenclature

CC: corpus callosum
DBS: deep brain stimulation
PD: Parkinson's disease
ROI: region of interest
DWI: diffusion-weighted imaging
FA: fractional anisotropy
MD: mean diffusivity
RD: radial diffusivity
MRI: magnetic resonance imaging
MPRAGE: magnetization-prepared-rapid-acquisition-gradient-echo
TR: repetition time
TE: echo time
FOV: field of view
SAR: specific absorption rate
AAL: AUTOMATED Anatomical Labeling
GPi: globus pallidus pars interna
STN: subthalamic nucleus
STR: striatum
SN: substantia nigra
ATAG: atlas of the basal ganglia
DN: dentate nucleus
SNR: signal-to-noise ratio
CNR: contrast-to-noise ratio

## 1 Introduction

High-frequency deep brain stimulation (DBS) is a surgical technique, which sends electrical pulses to the brain through permanently implanted electrodes. Electrodes are placed using stereotactic methods based on imaging studies combined with real-time intraoperative imaging or electrophysiology (Mädler and Coenen, 2012). This technique is used to treat, among other refractory diseases, the debilitating motor symptoms of advanced Parkinson's disease (PD). DBS is typically offered when a patient's symptoms are not well controlled with medication alone. The procedure's therapeutic effects involve modulation of basal ganglia circuits, although the exact mechanisms of action remain unknown (Okun, 2014). Despite DBS becoming a widespread treatment, uncertainties remain regarding which circuits are affected, which neural populations need to be targeted, and what is the most efficacious stimulation protocol. (Lozano et al., 2019)

Diffusion-weighted imaging (DWI) can be used to study brain microarchitecture in PD patients (Zhan et al., 2013). Specifically, this advanced imaging technique uses the diffusion of water molecules to generate contrast in MRI images and can be used to estimate white matter tracts of the brain (Coenen et al., 2012). The most commonly used values for clinical studies include fractional anisotropy (FA), mean diffusivity (MD), and radial diffusivity (RD). Changes in these values may indicate changes in neuronal pathology and therefore, allow for the quantitative assessment of white matter fibers in selected brain regions with only a few summarizing values (Feldman et al., 2010; Zheng et al., 2014; Vaillancourt et al., 2009). FA is a scalar value between zero and one that describes the degree of anisotropy of a diffusion process, the degree of which is thought to reflect fiber density, axonal diameter, and myelination. Similarly, MD is a summary measure of the diffusion properties of a given voxel, and equivalent to the estimated ADC over three orthogonal directions. Lastly, RD is a measure of the two small axes tensors which are averaged to produce a perpendicular diffusivity. Together, these measures are thought to reflect pathological processes including membrane integrity, myelination, and axonal density (Özarslan et al., 2005). DWI, therefore, has the potential to serve as a tool capable of monitoring changes in specific white matter tracts thought to be relevant to PD (Chen et al., 2018).

Currently, post-operative imaging of patients with implanted electrodes is most commonly used to confirm electrode location and screen for radiographic evidence of complications (Saleh et al., 2016). Clinical application of DWI post-DBS is currently limited due to the production of susceptibility artifacts from lead placement (Pollo et al., 2004). As a result, the few studies that have performed postoperative imaging analysis in DBS patients are limited by small sample sizes, a lack of clinical application, or a lack of DWI (Saleh et al., 2016; Coenen et al., 2011). This gap in investigation may be attributed, in part, to concerns regarding the safety of postoperative DWI or to a general perception that susceptibility artifacts will render post-operative imaging useless. Therefore, there is neither a consensus on the most efficient post-operative imaging methodology, nor is there any standardization for automatic or manual analysis (Saleh et al., 2016). No study to date has specifically analyzed diffusion scalars or the resulting tractography in postoperative DBS patients imaged with a clinically feasible protocol.

In the present study, we aim to evaluate the feasibility of postoperative DWI analysis and white matter reconstruction, using patients who have been scanned with clinically feasible scanning parameters. We set out to retrospectively explore and quantify the effects of susceptibility-induced artifacts on DWI metrics in patients imaged after DBS implantation. Quantification of these effects serves as a step towards our ability to better understanding the anatomical white matter changes that occur in response to treatment, ultimately providing insight into the therapeutic mechanisms behind DBS.

## 2 Methods

This study was performed under the approval of the local institutional review board. All patients consented to pre and post-operative imaging as part of routine clinical practice.

### 2.1 Image acquisition

All patients underwent preoperative magnetic resonance imaging (MRI) for pre-surgical evaluation and to plan the DBS procedure. All pre-operative neuroimaging of patients was performed under a standardized general anesthetic protocol, in order to eliminate movement. A standardized protocol of induction medications, percent sevoflurane concentrations, hemodynamic parameters (i.e. mean arterial pressure), and respiratory parameters (i.e. end tidal CO2, respiratory rates, oxygen concentration, tidal volumes) was instituted for all scans. Based on clinical indications, eight patients were scanned pre-operatively on a 3 T Philips Achieva scanner with an 8-channel head coil, while one patient was scanned pre-operatively on a 1.5 T Philips Achieva scanner using a birdcage RF transmit-receive coil. For the purpose of DBS trajectory planning, patients underwent a T1-weighted scan, a T2-weighted scan, and proton density weighted images. T1-weighted structural scans were based on a magnetization-prepared-rapid-acquisition-gradient-echo (MPRAGE). Imaging parameters of the MPRAGE sequence included an isotropic resolution of 1.0 × 1.0 mm with a slice thickness of 1.0 mm, repetition time (TR) = 7.0 ms, echo time (TE) = 3.0 ms, flip angle = 9°, matrix size = 512 × 512, field of view (FOV) = 25 cm. T2-weighted and proton density weighted images were acquired using a dual spin echo with turbo spin echo sequence for delineating targets of DBS. Imaging parameters of this sequence included an isotropic resolution of 2.0 × 2.0 mm with a slice thickness of 2.0 mm, TR = 5.24 s, TE1 = 16 ms, TE2 = 80 ms, matrix size = 256 × 256, FOV = 24 cm. Diffusion images were acquired for tractography. Patient's then underwent targeted DBS surgery within 2–3 weeks after image acquisition.

After surgery, additional post-operative imaging was acquired to verify appropriate electrode position. As part of standard clinical practice, post-operative imaging was acquired within 24 h of the surgery, and was taken on average 15 days after the pre-operative scan (Table 1). All post-operative neuroimaging was performed in the absence of anesthesia, as there was no clinical justification or ethical rationale for the post-operative induction of anesthesia given the primary goals of confirming electrode location and assessment for any postoperative complications. Patient demographics can be observed in Table 1.

**Table 1:** Time between image acquisition and post-operative scan (surgery date), as well as patient demographics.

	*n* = 9
Days between pre and post-operative scan	18.3 ± 5.0
Age (y)	67.6 ± 7.3
Sex (M:F)	6:3
Disease duration (y)	8.1 ± 2.5
Target (GPi:STN:VIM)	6:2:1
Laterality (B:L:R)	9:0:0
Preoperative scan (3 T:1.5 T)	8:1

All nine post-implantation scans were acquired on a 1.5 T Philips Achieva scanner using a birdcage RF transmit-receive coil within 24 h of implantation of bilateral DBS electrodes (3387 or 3389 lead, Medtronic; Minneapolis, MN) and an anterior chest wall implantable pulse generator (Activa PC, Medtronic; Minneapolis, MN). To ensure patient safety, SENSitivity encoding factor was turned off, and the DBS device was turned off. The post-operative scans followed an identical imaging protocol as the pre-operative imaging, with the specific absorption rate (SAR) values reduced to 0.1 W/kg or less to adhere to Medtronic safety guidelines (http://manuals.medtronic.com/manuals/mri/region) for imaging patients with DBS systems. While anatomical scans were required as part of each patient's clinical care, the diffusion sequences were added for purposes of this study. Imaging parameters for all 3 T preoperative and 1.5 T post-operative DTI scans can be seen in Table 2.

**Table 2:** Pre- and post-operative DTI acquisition parameters for 8 of the 9 subjects (note that one subject was scan pre-operatively using the 1.5 T Phillips Achieva scanner).

DTI Acquisition Parameters	Pre-operative	Post-operative
Scanner	3 T Philips achieva scanner	1.5 T Philips achieva scanner
Anesthetic protocol	Sevoflurane	None
Field strength (T)	3	1.5
Directions	32	32
*b*-value	800	800
TR (ms)	12,000–14,000	25,000–28,000
TE (ms)	80	145
FOV (cm)	24	24
Matrix	120 × 120	120 × 120
Voxel	2 × 2 × 2	2 × 2 × 2
Parallel factor	2	2
Acquisition time (min)	~15	~25

### 2.2 Image pre-processing

Diffusion data underwent motion and eddy current correction using the diffusion MRI *Artefact Correction In Diffusion* toolbox (http://www.diffusiontools.com/). To minimize eddy current and effects of motion, all DWI were registered onto the b0 image using a co-registration function utilizing affine-transforms. Larger transforms were required for the post-operative images to mitigate the head motion that occurred due to a lack of anesthesia. Pre- and post-operative FA, MD, and RD maps were created using Camino Software (http://camino.cs.ucl.ac.uk/). Scalar DTI values for each region within each patient were calculated by averaging the FA, MD, and RD values within each ROI individually, and were calculated for both pre- and post-operative scans.

### 2.3 Region of interest selection

To analyze whole-brain structures, both pre- and post-operative FA, MD, and RD maps for each individual subject were partitioned into 116 ROIs according to the Automated Anatomical Labeling (AAL) atlas (Mestres et al., 2000). Additionally, bilateral globus pallidus pars interna (GPi), subthalamic nucleus (STN), striatum (STR), and substantia nigra (SN) were registered to each patient using the data sets available from the atlas of the basal ganglia (ATAG) project (Keuken and Forstmann, 2015). Similarly, atlas registration of the dentate nucleus (DN) was performed for each patient using the quantitative susceptibility mapping based on the dentate nucleus atlas developed by (https://can.ucr.edu/software.html) HYPERLINK " He et al. (2017). The FreeSurfer analysis suite (http://surfer.nmr.mgh.harvard.edu), was used to automatically parcellate cortical regions of T1-weighted images based on patient anatomy, specifically for the extraction of the region of the corpus callosum (CC), since this region is not included in the AAL nor the ATAG atlases. A total of 127 structures were delineated from each subject.

### 2.4 Signal-to-noise and motion analysis

Measurements of signal-to-noise ratio (SNR) and contrast-to-noise ratio (CNR) were obtained and averaged across volumes of the artifact-corrected diffusion images. SNR was defined as the mean of the patient's gray matter voxels (Mean_GM_) over the standard deviation of air (Standard Deviation_AIR_):

(1) $$SNR=\frac{Mean_{GM}}{Standard\;Deviation_{AIR}}$$

Whereas CNR measurements were defined as the mean of the patient's gray matter voxels subtracted by the mean of the patient's white matter voxels, over the standard deviation of air:

(2) $$CNR=\frac{Mean_{GM}-Mean_{WM}}{Standard\;Deviation_{AIR}}$$

### 2.5 Artifact segmentation and analysis of effected regions

Susceptibility-induced artifacts were manually segmented by visual inspection of the regions containing signal dropout on the b0 images for each patient. Previously defined ROIs that included these manual segmentations were considered to be “affected” and the remainder of structures, which did not include the electrode artifact were labeled “unaffected.” Affected structures were grouped and analyzed against unaffected structures for all diffusion metrics.

Head motion parameters were extracted from the preprocessing motion-correction step using MATLAB. The severity of motion between both the pre- and post-operative scans were analyzed by averaging the amount of translation and rotation in the *x, y*, and *z*-directions, and compared between scans.

### 2.6 Regional statistical analysis

A within-patient analysis was performed to quantify the differences between preoperative and postoperative images. Statistical differences between pre- and post-operative FA values were calculated within each ROI of the AAL atlas, STN, GPi, SN, and CC for each subject. A two-sample Kolmogrov-Smirnov test was performed to test whether or not the voxels within each ROI contained a similar distribution within each ROI. If the two-sample Kolmogorov–Smirnov test rejected the null hypothesis, a paired Wilcoxon Signed Rank test was used to assess whether the population mean ranks differ. If the samples were from a similar distribution, a paired *t*-test was used in order to determine whether the mean of the variables were the same for the two groups, and a confidence interval for the true population was determined. Analogous subgroup analyses were performed specifically for affected ROIs and unaffected ROIs in order to directly evaluate the effect of implant artifacts. Particular attention was paid to regions of the SN and CC, given their previously described importance in the assessment of PD (Coenen et al., 2012).

### 2.7 Field strength analysis

The distribution of FA, RD, and MD values for all regions analyzed (AAL atlas, STN, GPi, SN, and CC) were plotted for comparison for each patient's pre- and post-operative scans (Fig. 2). Values were examined for differences specifically caused by the changes in field strength.

### 2.8 Tractography

In order to resolve the issues of crossing fibers, a Markov Chain Monte Carlo sample was built using a Bayesian Estimation of Diffusion Parameters (BEDPOSTX) as implemented by FSL's diffusion toolbox. Where the diffusion coefficient is modeled using a Gamma distribution, with a maximum of two fibers per voxel. Probabilistic tractography of the nigrostriatal, dentate-rubro-thalamic, and hyperdirect pathways were performed using FSL's Probtrackx 2.0. For all subjects, each voxel within the defined seed regions was seeded with 5000 streamlines that migrated according to local probability density functions for both pre- and post-operative DWI sequences. Streamlines seeded from the striatum (STR), which contacted the substania nigra (SN) were retained as estimates of the nigrostriatal pathway. Similarly, those which contacted the dentate nucleus (DN) from the thalamus were retained as estimates of the dentato-rubro-thalamic pathway. And those which contacted the subthalamic nucleus (STN) from the precentral gyrus were retained as estimates of the hyperdirect pathway (Behrens et al., 2007).

The default settings of Probtrackx 2.0 were used with a setting of 5000 samples per voxel, a step length of 0.5 mm, a curvature threshold of 0.2, and a “loopcheck” to ensure that no tracts were included that doubled back on themselves. The “classification target” option was chosen, in which the STR, thalamus, and cortical areas were set as the seed masks; and the SN, DN, and STN were set as classification targets, respectively. A value representing the number of streamlines propagating from the seeded regions to the classification targets was calculated for each voxel in this manner. As such, the resulting output map represents the robustness of the fiber tract as described by the probability of its existence in a particular voxel between the seed region and classification target (Behrens et al., 2003).

Two previously-described variables of connectivity were then calculated to represent the robustness and likelihood of a tract reconstruction (Theisen et al., 2017):

(3) $$index\;of\;connection\;probability=\;\frac{\sum probability\;of\;connection\;to\;the\;classification\;target}{\sum total\;number\;of\;voxels\;in\;seed\;mask}$$

(4) $$streamline\;density=\;\frac{\sum number\;of\;nonzero\;voxels\;in\;resultant\;map}{\sum total\;number\;of\;voxels\;in\;seed\;map}$$

**Fig. 1:**
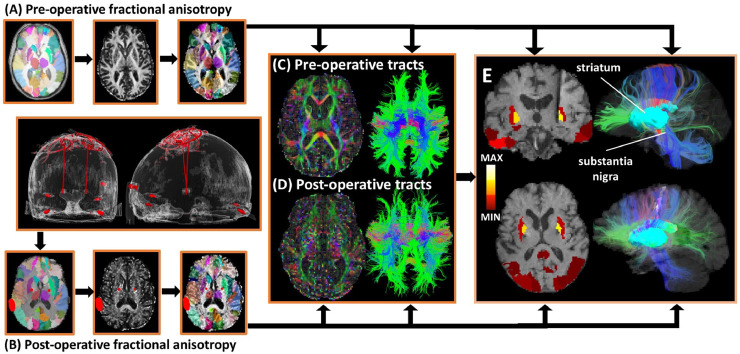
Flowchart of pre and post-operative analysis. The T1 image of the single-subject template in subject space (A) was registered in the pre and post-operative b0 image of each subject in their native space (B) with the transformation T. The atlas labels were transferred to the native space with the transformation T. (C) and (D) show both the pre and post-operative FA map and tractography results in native space, respectively. Pre and post-operative DTI scalars and tractography were compared and analyzed for differences (E).

Probability values, generated by Probtrackx 2.0, were analyzed within the termination mask (STN, DN, and SN) of each patient's individual tractography results on a voxel-by-voxel basis. These measures were calculated for both pre- and post-operative scans for the three aforementioned tracts in all subjects. Aside from the index of connection probability and the streamline density, a voxel-wise significance test was performed to the scalar values of pre- and post-operative connection probability values within each target ROI (STN, DN, and SN) using a two-tailed heteroscedastic *t*-test. Findings of a *p* value > 0.05 were considered significantly similar. A pipeline of the post-operative analysis can be seen in Fig. 1.

## 3 Results

### 3.1 Motion and signal-to-noise

All subjects had more motion in the un-anesthetized postoperative scan, than in the anesthetized preoperative scan. On average, motion artifacts of the postoperative scans were 3.43 times larger than that of the motion distortion in the preoperative scans, with postoperative scans having a translation of 0.56 ± 1.06 and preoperative scans having a translation of 0.16 ± 0.35 during motion correction (Appendix A.1).

SNR and CNR of the postoperative scans were greatly reduced, with the postoperative SNR (8.26 ± 0.54) being approximately 300 times less than that of preoperative SNR (324.56 ± 255.35) (Appendix A.2). Similarly, the postoperative CNR (−0.01 ± 0.22) was found on average to be 35 times less than that of the preoperative CNR (12.29 ± 10.08) (Appendix A.3).

### 3.2 Diffusion scalar results

**Fig. 2:**
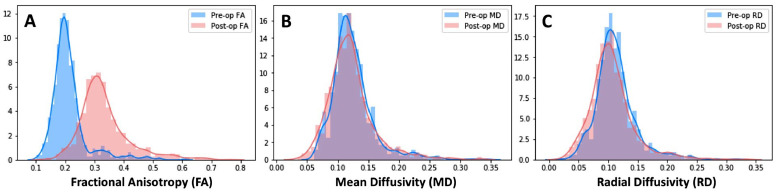
Distribution of pre- and post-operative of fractional anisotropy (A), mean diffusivity (B), and radial diffusivity (C) values across for 8 subjects for all 123 structures, excluding the subject scanned pre-operatively at 1.5 T.

Post-operative FA, throughout the brain were found to be consistently higher across all subjects, whereas MD and RD values seemed to have similar distributions between pre and post-operative scans. Whereas all postoperative values of FA increased, 47.15% of MD and 57.99% of RD values were found to be lower postoperatively relative to the preoperative scan (Fig. 2).

FA was found to be postoperatively higher relative to the preoperative values for all ROI across subjects: 95.56% of structures were found to have different underlying distributions in FA. Of the structures with different distributions, the Wilcoxon rank test determined that 2.63% came from distributions with equal medians (*p* > 0.05). Of the 4.44% of structures with similar distribution, 77.08% of these were found to have equal means (*t*-test *p* > 0.05). For RD, 93.15% of structures were found to have different underlying distributions with approximately half of all structures (58.61%) having relatively lower values in the postoperative scan when compared to the preoperative. Of the structures with different distributions, 26.27% had equal medians (*p* > 0.05), and of those with similar distributions, 98.65% had equal means (*t*-test *p* > 0.05). For MD, 93.15% of structures were found to have different underlying distributions with 47.50% of regions having MD values lower in the postoperative scan. Of the regions containing different distributions, 27.97% had equal median distributions (*p* > 0.05), and of the structures with similar distribution, 94.59% contained equal means (*t*-test *p* > 0.05). The results of the voxel-wise scalar analysis for the SN and CC for all patients, as well as the percent change for statistically different structures, are displayed in Table 3.

**Table 3:** Scalar analysis for diffusion metrics displayed for all subjects. If the two-sample Kolmogorov–Smirnov test determined that the values were drawn from a similar distribution, an unpaired *t*-test was performed. If the distribution was different, a Wilcoxon Signed Rank test was performed. *P*-values are reported for left and right SN, and CC for all patients across metrics.

Case no.	FA, MD, and RD analysis in the SN and CC across subjects					
	FA		RD		MD	
	*T*-test (*P*-value)[CI]	Signed-rank (*P*-value)	*T*-test (*P*-value) [CI]	Signed-rank (*P*-Value)	*T*-test (*P*-value) [CI]	Signed-rank (*P*-value)
1.SN L	0.957 [−0.054,0.052]			0.198		0.013
SN R		0.350		0.084		0.047
CC		0.531		0.119		<0.001
2.SN L		<0.001		<0.001		0.017
SN R		<0.001		0.030		0.603
CC		<0.001		<0.001		0.005
3.SN L		0.196		0.423		<0.001
SN R		0.835		0.026		<0.001
CC		0.591		0.630		0.245
4.SN L		0.003	0.062 [−0.004,0.153]		0.292 [−0.032,0.107]	
SN R		0.016		0.021		0.305
CC		<0.001		<0.001		<0.001
5.SN L		0.756		0.101		0.035
SN R		0.357		0.470		0.146
CC	0.567 [−0.050,0.027]		0.038 [0.005,0.182]			<0.001
6.SN L	0.053 [−0.001,0.123]		0.034 [−0.353,−0.14]		0.064 [−0.319,0.009]	
SN R	0.010 [−0.121,0.011]		0.556 [−0.194,0.107]		0.323 [−0.214,0.071]	
CC	0.743 [−0.045,0.032]		0.550 [−0.114,0.061]			0.079
7.SN L	0.051 [−0.071,0.035]		0.223 [−0.171,0.040]			0.493
SN R	0.643 [−0.035,0.056]			0.556		0.940
CC		0.036	0.056 [−0.186,0.002]			0.094
8.SN L		0.002		0.317		0.550
SN R		0.820		0.767		0.189
CC		<0.001		<0.001		<0.001
9.SN L		0.016		0.135		0.350
SN R		0.006		0.128		0.625
CC		<0.001		0.056		0.166

### 3.3 Regional susceptibility artifact results

**Fig. 3:**
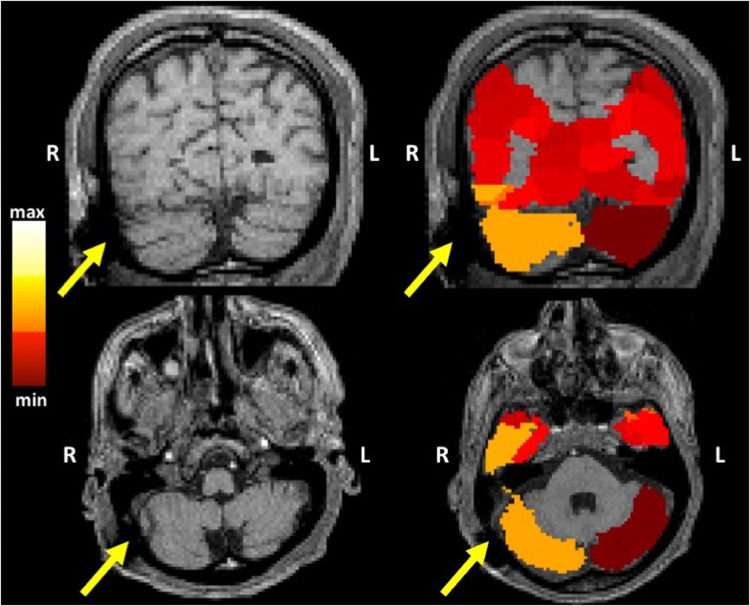
Regional differences in FA, those with higher differences are shown to clearly intersect with susceptibility artifact. The yellow arrow indicates signal dropout caused by the connection wire between the electrode and the implantable pulse generator.

Approximately 20.05% of ROIs across all patients contained susceptibility artifacts. Artifact was found to be generated by the connector to the DBS extension, resulting in signal loss observed over the occipital lobe (Fig. 3). Of the structures were which affected by artifact, 97.75% were found to have different distributions in FA as tested by the two-sample Kolmogorov-Smirnov goodness-of-fit hypothesis test. Only 1.99% of affected structures were found to have similar means (*p* > 0.05), with 60% of the structures having similar distributions coming from equal means (*t*-test *p* > 0.05). For MD 98.65% of structures had different distributions, 26.92% of which had similar means (*p* > 0.05), with only 3 total structures having both similar distributions and equal means (*t*-test *p* > 0.05). For RD 97.75% had different distributions, 25.84% of which had similar means (*p* > 0.05), with the 5 total structures of similar distributions having equal means (*t*-test *p* > 0.05).

### 3.4 Tractography results

**Fig. 4:**
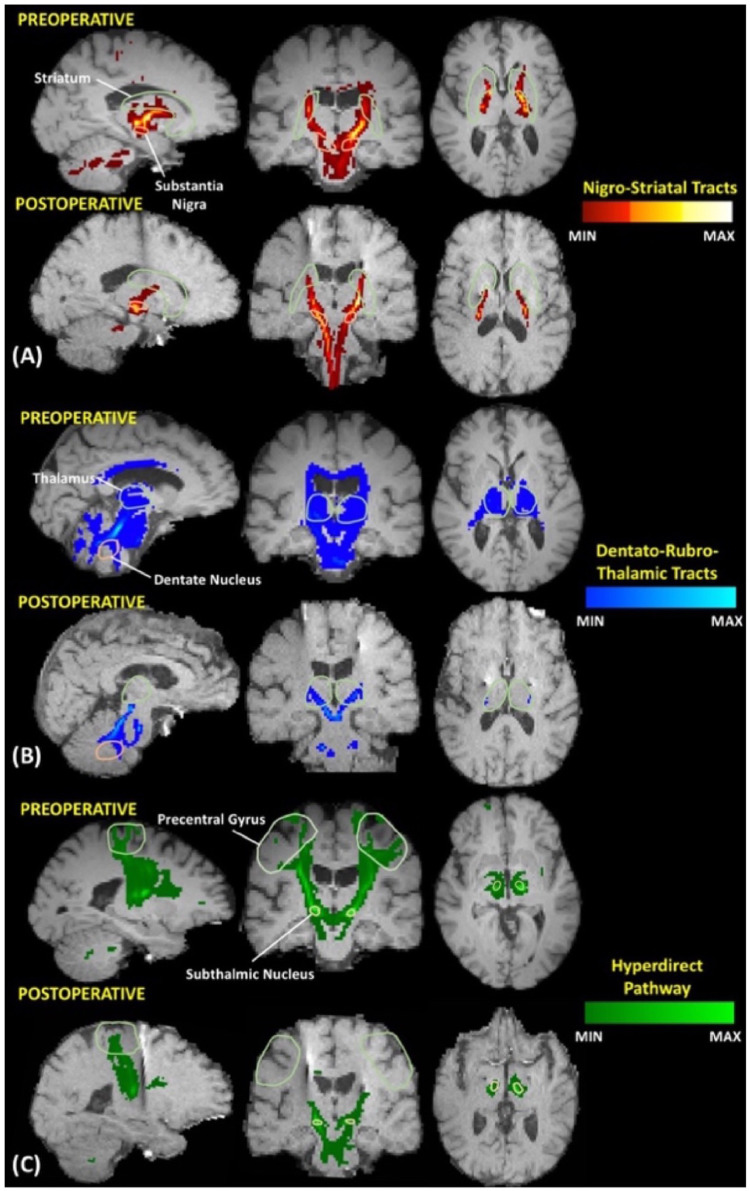
Pre and post-operative tractography of the dentato-rubro-thalamic tracts of the hyperdirect pathway, and nigrostriatal tracts for a single subject (*subject 3*).

The index of connection probability and streamline density average across subjects for both the pre-op and post-operative scans, as well as the percent change can be seen in Table 3. Detection of the nigrostriatal pathway appeared to be the least affected, with 6 of the 9 patients having a significantly similar reconstruction of the pathway in either the left or right hemisphere (*p* > 0.05). This finding is in concordance with lower percent changes in the index of connection probability and streamline density in the nigrostriatal pathway. For the dentato-rubro-thalamic and the hyperdirect pathways, only three subjects demonstrated significantly similar tract reconstructions (Table 4(C)). Visualization of pre and post-operative tractography within a single subject (scanned pre-operatively at 3 T and post-operatively at 1.5 T) are displayed in Fig. 4.

**Table 4:** Tractography results. (A) The connection probability index, as well as the maximum and minimum probability averaged across all seven subjects, for the nigrostriatal, dentate-rubro-thalamic and hyperdirect pathway (B) The streamline density averaged across subjects (C) Statistical results for the connection probability for all subjects.

Index of connection probability^a^	Nigro striatal		Dentato-Rubro-Thalamic		Hyperdirect pathway	
	Pre-op	Post-op	Pre-op	Post-op	Pre-op	Post-op
	204.8	72.2	221.8	23.3	7.9	0.76
Percent change	64.8%		89.5%		90.4%	
(A) **Streamline density**^b^						
	Pre-op	Post-op	Pre-op	Post-op	Pre-op	Post-op
	71.5	26.3	80.0	36.6	36.1	4.2
Percent change	63.2%	54.3%	88.4%			
(A) ***T*-test of connection probability**	**Nigrostriatal**		**Dentato-Rubro-Thalamic**		**Hyperdirect pathway**	
	**Left**	**Right**	**Left**	**Right**	**Left**	**Right**
**Subject 1**	0.606*	0.179*	<0.001	<0.001	<0.001	0.013
**Subject 2**	0.106*	<0.001	<0.001	<0.001	0.001	<0.001
**Subject 3**	<0.001	0.311*	<0.001	<0.001	<0.001	<0.001
**Subject 4**	0.0248	<0.001	<0.001	<0.001	<0.001	–
**Subject 5**	0.066*	<0.001	<0.001	<0.001	<0.001	<0.001
**Subject 6**	0.818*	0.690*	<0.001	0.616*	0.878*	<0.001
**Subject 7**	0.002	<0.001	<0.001	<0.001	–	<0.001
**Subject 8**	<0.001	0.027	<0.001	<0.001	0.072*	0.501*
**Subject 9**	0.117*	0.709*	<0.001	<0.001	3.234 e −7	0.094*

a Mean number of samples that generate streamlines that reach the SN per seeded STR voxel, DN per seeded thalamus voxel, and STN per seeded cortical seed voxel, averaged across subjects from pre to post-operative.

b Percentage of voxels in the STR that generate one or more streamlines that reach the SN.

⁎ *p* > 0.05.

## 4 Discussion

### 4.1 Safety in post-operative diffusion imaging

Despite potential hazards involved when exposing a patient with DBS to MRI, MRI provides the benefit allowing both electrode and artifact, and the intended target to be visualized on the same image, and is therefore preferable for verification of lead location in a clinical setting (Zrinzo et al., 2011; Hariz et al., 2003; Petersen et al., 2010; Tisch et al., 2007). All patients in this study were able to safely undergo MRI scans, including diffusion sequences, as the field strength and SAR were restricted according to manufacturer- and FDA-delineated parameters of 1.5 T and 0.1 W kg^-1^, respectively. While these parameters are specific to the system used in this study, device manuals typically provide instructions on how MRI can be conducted safely after implantation of DBS electrodes (Medtronic, 2007) and are relatively comparable. Ultimately, the number of reported MRI-related adverse events is surprisingly low, with the only adverse event reported in regard to 1.5 T scanning to be related to an implant malfunction (Tagliati et al., 2009). While advancements have been made towards demonstrating the feasibility of low SAR imaging of DBS patients at 3T using patient-derived FEM models with realistic device configurations (Kazemivalipour et al., 2019). However, diffusion properties of tissues are independent of field strength, since water diffusion is not influenced by the magnetic field, making diffusion imaging methods well-suited for research studies which combine data from varying field strengths (Alexander et al., 2006). A study by Hunsche et al. performed on seven healthy volunteers, compared values of diffusivity and FA using varying field strengths of 1.5 and 3.0 T and did not yield any significant differences in FA and MD (Hunsche et al., 2001). In the current study, however, it was observed that post-operative FA values at 1.5 T were higher by an offset of about 1.5 times than that of the pre-operative FA values scanned at 3 T (Fig. 2). This difference is likely attributable to the sensitivity of DTI measurements to the lower image SNR and increased motion associated with the postoperative scans.

### 4.2 Motion, SNR, and CNR analysis

In typical MRI imaging, the SNR can be approximated as proportional to magnetic field strength. Due to the change in field strength, we confirmed the assumption that SNR and CNR of post-operative DWI decreases as a result of the artifact. Low SNR in DTI may cause estimated eigenvalues and anisotropy to be biased, leading to an overestimation of diffusion anisotropy and an overall increase in scalar values. Both SNR and CNR were greatly reduced in the post-operative scans. Low SNR may contribute to the sudden observed changes in post-operative FA, as low SNR has been found to increase noise and decrease contrast between gray and white matter in DTI-derived FA images (Farrell and Landman, 2007). While this serves as a limitation for this study, it also increases its clinical applicability, as most pre-operative imaging necessitates a 3 T scan, while post-operative imaging is limited to 1.5 T. The data presented herein emphasizes the need to account for the effects of experimental noise in assessing anisotropy of individual eigenvalues of the diffusion tensor, particularly when comparing measurements from different field strengths, where considerable changes in SNR occurs. Knowledge of the SNR and CNR measurements in white matter regions can be used to eliminate noise bias and estimate true anisotropy (Mukherjee et al., 2002). The affects of field strength between 3 T and 1.5 T on diffusion metrics in normal adults have been well described in previous publications, and have described that while in theory diffusion metrics should not be influenced by field strength, nonlinear changes do occur (Polders et al., 2011;Kosior et al., 2007; Alexander et al., 2006; Hunsche et al., 2001). Specifically, a within-site examination of diffusion values found that FA can generally be considered reproducible within each site (Helmer et al., 2016).

In addition to experimental noise, motion, whether physiological or patient, is a major source of error in MRI and has been shown to significantly impact estimation of FA values (Middleton et al., 2014). As observed by the motion analysis performed on pre- and post-operative DWI images, post-operative motion is significantly larger when compared to that of the anesthetized pre-operative scan. Even small amounts of motion have been shown to cause large signal variations, affecting the reproducibility and precision of DTI data and subsequently derived MD and FA maps (Tijssen et al., 2009). In past studies, retrospective head motion correction has been shown to alter the estimation of scalar metrics used in clinical DWI studies, along with across-session reproducibility errors (Kreilkamp et al., 2016). Taking into account head motion variation is essential for a more accurate estimation of DWI measures, particularly when comparing anesthetized versus un-anesthetized scans.

Similar to the limitation of field strength, this study reflects what most centers would be able to accomplish with postoperative imaging and puts an emphasis on clinical feasibility and generalizability across centers. When attempting post-operative DWI, the effects of field strength, coil geometry, and motion correction, should all be considered as major contributors to differences between the pre- and post-operative scans.

### 4.3 Role of diffusion imaging in Parkinson's disease

Careful monitoring of PD patients has led to important developments in our understanding of the disease and intervention. At the point of clinical expression, it is approximated that nearly half of the dopaminergic cells in the SN are lost, allowing us to use DWI to provide an indirect measure of degeneration within the SN, as the cell loss alters microstructural integrity and diffusion of water molecules during disease progression (Vaillancourt et al., 2009). More specifically, PD has been associated with characteristic regional patterns of degradation in the SN, putamen, and thalamus, as well as the motor, premotor, and supplementary motor cortices. A significant obstacle in understanding the mechanism of action of DBS is the lack of a quantitative understanding of the extent and influence on the neural elements involved. It is believed that changes in the underlying dynamics of stimulated brain networks may be the main cause for the therapeutic results observed after DBS. (Kazemivalipour et al., 2019) These regional changes can be detected by DTI and are associated with PD severity, varying with main motor PD subtypes (Zhan et al., 2012). For example, the hyperdirect pathway is a connection to the basal ganglia which is related to the initiation, execution, and termination of voluntary limb movement (Nambu et al., 2002). DTI tractography has been used to show that the hyperdirect pathway connects the STN with cortical motor areas, and may be readily reconstructed in patients with advanced PD (Muller et al., 2019). These pathways and regions, rebuilt by DTI, can help further elucidate the mechanisms of DBS and guide therapy (Chen et al., 2018). Although little is known about the mechanisms of DBS, it appears that there is direct involvement of axonal fibers, rather than gray matter, suggesting that both local and large networks are being targeted (Ashkan et al., 2017). Recently, modulation of specific brain regions and white matter tracts has been associated with improvements in particular PD subtypes and symptomologies (Akram et al., 2017). These findings merit efforts towards the longitudinal tracking of postoperative DBS patients in order to associate white matter network integrity with the long-term effects of DBS.

### 4.4 Diffusion scalar analysis

Although diffusion metrics within all structures appeared to be affected by susceptibility artifacts, it is discernible that some regions are significantly more affected than others, and that MD and RD were less affected, having similar distributions when comparing the pre and postoperative scans (Fig. 2). Larger changes in FA are to be expected as this particular diffusion scalar measurement is most sensitive to microstructural change, as well as susceptibility artifact (Alexander et al., 2011). Structures directly intersecting with the electrode artifact segmentation were affected approximately three times more than those which were not, including regions which were target sites for electrode implantation (GPi, STN, and VIM). This can be attributed to intense signal dropout. However, despite the large disparity in pre- and post-operative FA values, major regions related to PD remained to be unchanged, as can be viewed in Table 3, where a large portion of the regions of the SN and CC were significantly similar across subjects. Additionally, approximately half of the RD and MD values across all brain regions and across all subjects were found to have no significant change, indicating these scalar values are functional as biomarkers of disease progression for diffusion analysis of patients who have undergone DBS.

The ability to continue to visualize the microarchitecture of particular brain regions proves useful for longitudinal tracking of post-operative DBS patients, as it has been comprehensively suggested that diffusion measures within the SN and CC may prove useful for tracking disease progression (Menke et al., 2009). Specifically, it has been shown that FA has the ability to distinguish between healthy subjects and de novo PD individuals, particularly in the caudal region of the SN (Vaillancourt et al., 2009), whereas RD has been seen to increase in the corpus callosum, internal and external capsules, corona radiata, sagittal stratum, fornix, and cingulum (Guimarães et al., 2018). Previously, several studies including Menke et al. (2009), Vaillancourt et al. (2009), Schwarz et al. (2013), Lenfeldt et al. (2015), and others, have investigated diffusion measures within the SN as a potential biomarker for PD (Lenfeldt et al., 2015; Menke et al., 2009; Schwarz et al., 2013; Vaillancourt, 2009; Zhan et al., 2013). Therefore, proving our capability to continue to analyze post-operative diffusion metrics in these particular regions is significant. In the past, the use of DWI has been confounded by the presence of susceptibility artifacts from lead placement. We report for the first time the feasibility of post-operative diffusion analysis in nine patients with advanced PD. In this work, we establish measures of reliability of specified tracts in the postoperative setting for individual patients to determine whether or not a patient is eligible for longitudinal evaluation by DTI. The results of this study suggest the possibility of a follow-up analysis using advanced diffusion parameters and tractography on post-operative DBS patients, a process useful for the clinical correlation of DBS outcomes with anatomical biomarkers (Danielian et al., 2010).

### 4.5 Tractography

Three specific tracts were chosen for analysis, based on their relevance to PD and their history as an area of interest for DBS. Overall, it appears that structures not involving the cortex had the highest retention of tracts in postoperative imaging, with the nigrostriatal pathway being the best retained, followed by the DRT and the hyperdirect pathway (Table 4).

The nigrostriatal pathway is an important connection of the basal ganglia, which connects the SN with the dorsal striatum, and is particularly involved with control of motor behavior (Tritsch et al., 2012). This pathway has been shown to degenerate in patients with PD as dopaminergic connections are lost (Tan et al., 2015). We were able to successfully delineate nigrostriatal tracts both pre- and post-operatively in patients, with six out of the nine patients having significantly similar probability of connection in at least one hemisphere. Moreover, the largest retention in both index of probability and streamline density were retained for the nigrostriatal pathway (Tables 4(A) and (B)), which is notable as this measure has been shown to be promising in its ability to distinguish PD subjects from healthy controls, and has been indicated as a method useful for tracking longitudinal changes within individuals (Theisen et al., 2017). The retention of this measure in post-operative DBS imaging is of great interest, as we may continue to track streamline density as a disease classifier as patients respond to therapy.

Additionally, it has been shown that modulation of the DRT is most likely responsible for the physiological effect of DBS in the thalamic/subthalamic region that leads to alleviation of tremor (Coenen et al., 2011). The delineation of the white matter tract of the DRT may be important for neurosurgeons during DBS in patients for tremor control. Damage to this tract may be responsible for motor symptoms and may lead to movement disorders such as ataxia or tremor (Hana et al., 2016). In addition to the DRT, the hyperdirect pathway forms connections between several cortical areas and the STN (Jahanshahi et al., 2015). Both the DRT and hyperdirect pathway have been shown to be responsible in the pathophysiology of tremor (Coenen et al., 2011; Hammond et al., 2007).

While tracts of the DRT and the hyperdirect pathway were found to be statistically different (*p* > 0.05) pre- to post-operatively for the majority of patients, retention in streamline density and connection index were moderately achieved, and reconstruction of both the DRT and hyperdirect pathway proved to be feasible (Table 4 and Fig. 4). This may be attributed to dramatic postoperative increase in FA, when compared to MD and RD, creating a difficulty in fiber tracking, particularly in regions which intersected with electrode artifact. Therefore, with more advanced improvements in diffusion-based techniques and image post-processing, it is possible that the measures of streamline density and connectivity index may prove useful for tracking longitudinal changes in these motor pathways within patients (Theisen et al., 2017). Through quantification of the degree at which these metrics have been affected by artifacts, a new baseline may be recognized at which we can evaluate the degeneration and integrity of the tracts months, or years, after therapy has been administered.

### 4.6 Delineation of error source and regional susceptibility artifact affects

While the purpose of this study was to prove the feasibility of post-operative imaging, we aim to likewise quantify and identify sources of disruption that would contribute to the changes observed in the quality of post-operative DWI. Here we have distinguished three separate contributing factors which may all contribute to the loss of diffusion single including: differences in SNR/CNR, head motion, and electrode artifact. While we have listed above the potential pitfalls of these confounding factors, and the steps taken to correct for them when relating diffusion metrics to the resulting clinical outcomes, it is advantageous to distinguish how much of the error is a result of the acquisition parameters versus the electrode artifact. In the present study, susceptibility artifacts caused by DBS electrodes and the connection wires to the electronic pulse generator were manually segmented (Fig. 1). It was found that the majority of structures which intersected with these susceptibility artifacts for each particular subject had significantly altered FA, MD, and RD values across patients, in both distributions and means. This supports the notion that any region intersecting with signal drop-out due to the implanted DBS system cannot feasibly be reconstructed using DWI. This finding has not been elucidated by previous work regarding signal noise interference caused by susceptibility artifacts generated by implanted DBS systems. Artifact delineation should be considered on a patient-to-patient basis, as configuration of the electrode artifact will differ due to the trajectory of the implanted electrode with respect to the scanning plane and the location of the connection to the extension wire (Saleh et al., 2016).

It is believed that the general increase in post-operative FA occurs globally in the brain due to the decrease in SNR, however the intense signal dropout of the metal artifact allows us to distinguish which regions are no longer able to be reconstructed due to distortions. (Saleh et al., 2016) This was particularly of interest in regions of the SN and CC, which are suitable biomarkers for disease progression (Lenfeldt et al., 2015). Therefore, this advancement towards accurate visualization of DWI is significant towards the critical interpretation of clinical outcome.

### 4.7 Limitations and future work

Limitations of the current study beyond the scope of field strength, motion artifacts, and post-operative SNR may also contribute to inaccuracies when defining post-operative diffusion metrics. Variation between pre- and post-operative scans may have been caused in part to wide-ranging scanner noise and inhomogeneity between sessions, as well as inaccuracies in post-processing such as misalignment of atlas registration (Buchanan et al., 2014). It should be noted that these errors created a systemic fairly consistent error across subjects, therefore inaccuracies due to inaccurate registration was consistent between patients. To improve upon this, a more accurate method of structure delineation such as manual segmentation or individualized cortical parcellation may be warranted. This is to be balanced with the time needed for manual segmentation, as well as the resolution of the DWI sequence. To eliminate noise created by aforementioned sources of error, a correction factor may be developed either through individual subject or group normalization. This may be done through methods of machine learning or other post-processing tools, and would drastically increase the accuracy of fiber tracking should it be applied to postoperative FA.

Additionally, all post-operative scans were performed using either a Medtronic 3389, or for thalamic stimulation, a Medtronic 3387 DBS electrode. Therefore, the results of this study may not be generalizable and should be repeated for all DBS lead models. This includes the application of varying safety regulations, since electrode artifacts may be variable for different manufacturers and scanning protocols.

Lastly, efforts should be made towards the inclusion of SAR reduction performance in clinical practice for the accessibility of safely imaging DBS patients at 3T.

### 4.8 Sample size and clinical outcomes

While the sample size for this dataset is small (*n* = 9), this dataset remains the largest study to date in humans, as post-operative DWI is rarely attempted or analyzed in post-DBS patients. This individualized study aims to elucidate the feasibility of longitudinal diffusion analysis for within-subject post-operative DBS patients, where variation in study population and an increase in numbers is desirable. Since the statistics generated in this feasibility study were not based on group analysis, the sample size does not have a direct effect on the results of the analysis. While beyond the scope of this study, we plan to scan these patients in a delayed fashion, several months and years into the future, to assess the ability of tracking differences in DBS patients longitudinally using DWI and correlate with the proceeding clinical outcomes.

Lastly, we understand that while correlation between DTI metrics and patient outcomes are important and the basis of future work, such analysis is beyond the scope of this study. The first step in bringing post-operative DBS imaging into clinical practice is to quantify the degree at which post-operative DWI are affected by artifacts as a means by which the feasibility of its use can be quantified. By assuming that no changes in anatomy have occurred within the <15 day time period between pre and post-operative imaging, changes in diffusion metrics can be attributed solely to field strength/scanner/post-implant changes. In the future, we hope to use the newly established baseline of DWI measures to individually track disease progression in DBS patients, which will then be correlated with clinical outcome. Through doing so, we may be able to distinguish structural changes which occur during DBS therapy, giving us a better understanding of how DBS works to reduce the symptoms of PD and of the pathophysiology of brain networks as a whole.

## 5 Conclusion

This pilot within-subject study demonstrates both the safety and feasibility of pulse sequences previously thought to be inaccessible to DBS patients, using a clinically reasonable timeframe. A unique dataset of nine post-operative DBS patients was analyzed using advanced probabilistic tractography to successfully reconstruct diffusion values of structures known to be associated with PD. Given the changes induced by changing field strength, SNR, and electrode artifact, it will be necessary to establish a new baseline if we are to longitudinally track diffusion metrics in post-operative DBS patients. Despite recent developments in our understanding behind the pathophysiology of PD, DBS, and its therapeutic effects, post-operative imaging for validation of brain changes associated with symptom improvement in PD will largely impact the field of functional neurosurgery. The benefits of developing a non-invasive method for analyzing postoperative DBS patients includes an accurate interpretation of its clinical effects, understanding the functionality of the stimulation targets, and ultimately elucidating the mechanisms underlying DBS.

## CRediT authorship contribution statement

**Jennifer Muller:** Conceptualization, Methodology, Software, Validation, Formal analysis, Investigation, Data curation, Writing - original draft, Writing - review & editing, Visualization, Supervision. **Mahdi Alizadeh:** Conceptualization, Investigation, Software, Validation, Writing - review & editing, Methodology. **Li Lucy:** Investigation, Methodology, Visualization, Writing - review & editing. **Sara Thalheimer:** Investigation, Data curation, Resources. **Caio Matias:** Software, Validation, Formal analysis, Resources. **Mohamed Tantawi:** Software, Validation, Formal analysis. **Miao Jingya:** Software, Validation, Formal analysis. **Mackenzie Silverman:** Conceptualization, Methodology, Software, Writing - original draft. **Zhang Veronica:** Software, Validation, Formal analysis, Writing - review & editing. **Yun Grace:** Software, Validation, Formal analysis. **Victor Romo:** Resources, Writing - review & editing. **Feroze B. Mohamed:** Investigation, Resources, Writing - review & editing, Project administration, Supervision. **Wu Chengyuan:** Conceptualization, Resources, Writing - review & editing, Project administration, Supervision.

## Figures and Tables

**Fig. A1:**
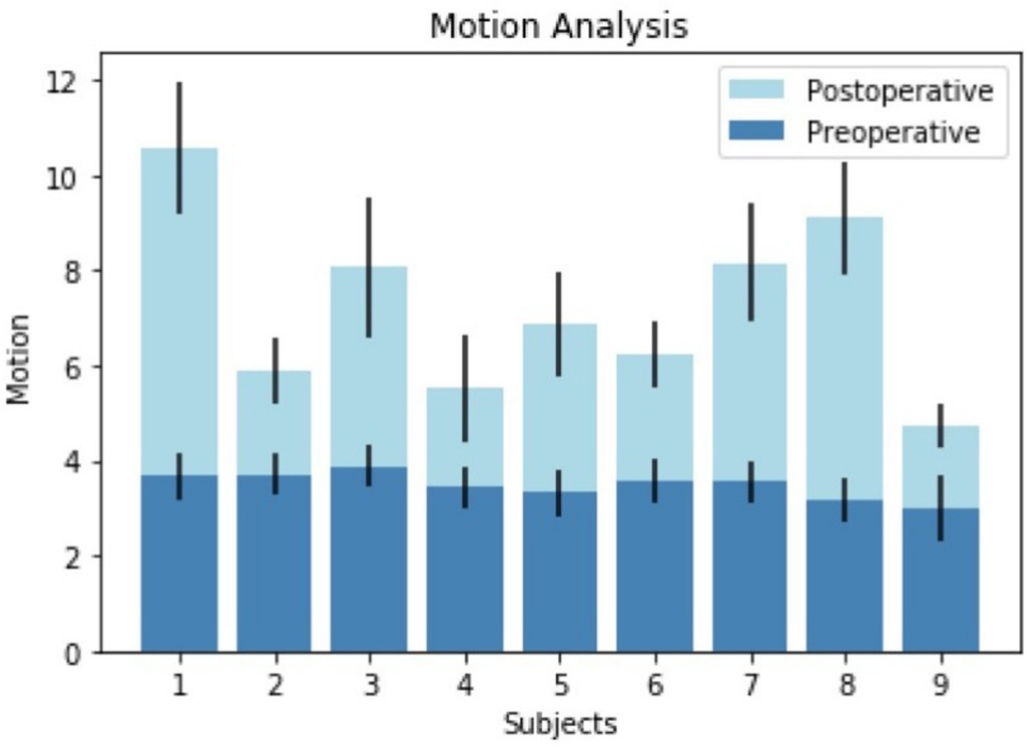
Results of the pre and post-operative motion correction analysis across subjects.

**Fig. A2:**
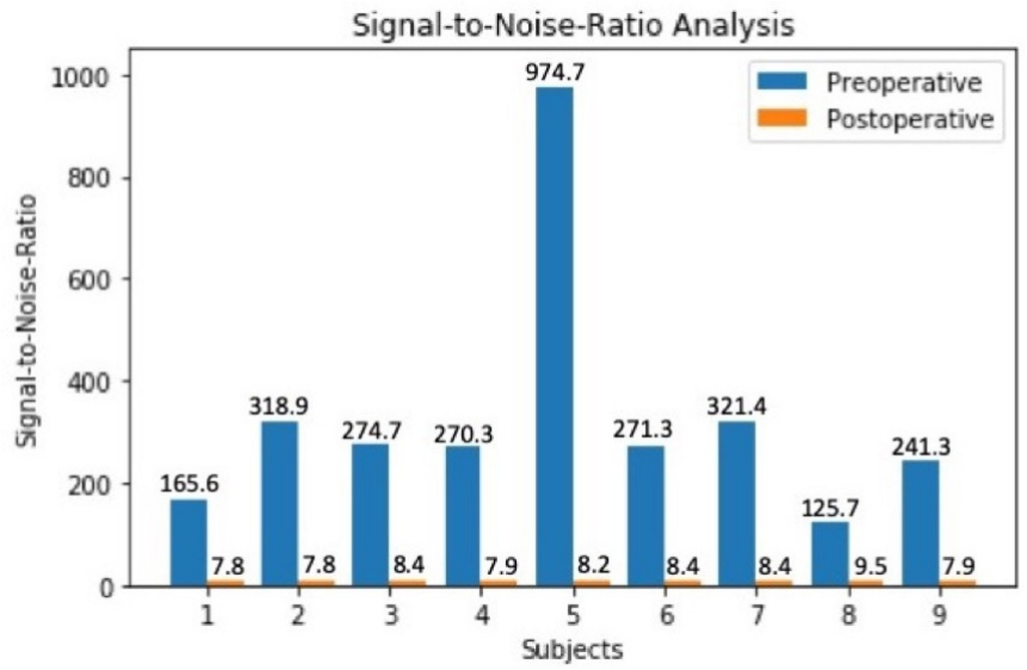
Results of the pre and post-operative signal-to-noise analysis across subjects, subject 5 scanned pre-operatively at 1.5 T.

**Fig. A3:**
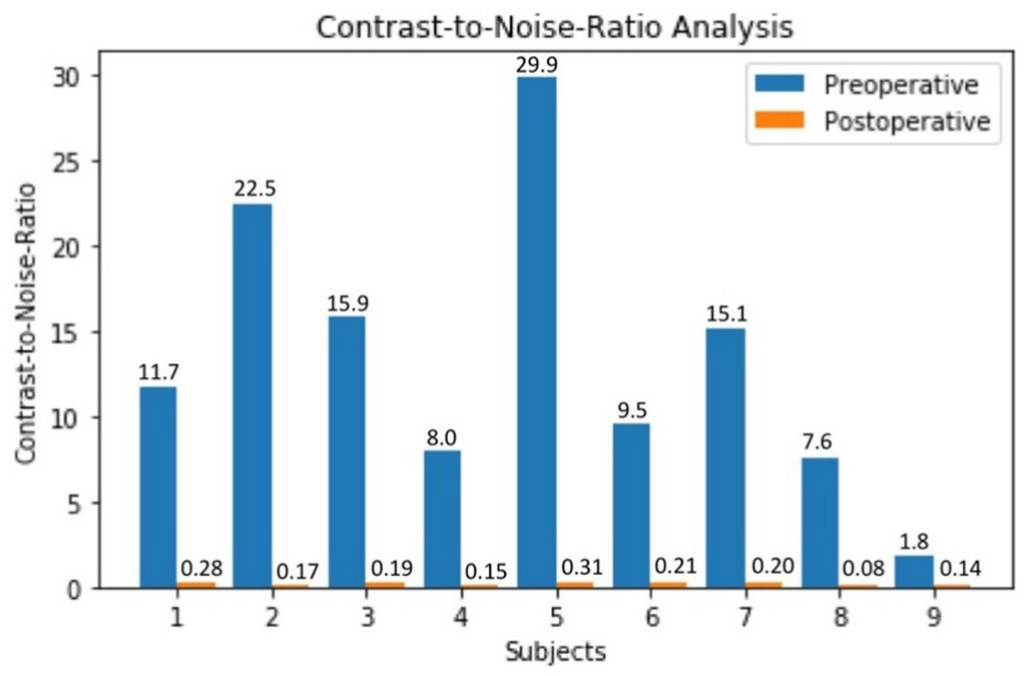
Results of the pre and post-operative contrast-to-noise analysis across subjects, subject 5 scanned pre-operatively at 1.5 T.

## Appendix A. Motion and signal analysis

### A.1. Motion correction analysis

Fig. A1.

### A.2. Signal-to-noise analysis

Fig. A2.

### A.3. Contrast-to-noise analysis

Fig. A3.
